# Integration of the Vision of People With Diabetes Into the Development Process to Improve Self-management via Diabetes Apps: Qualitative Interview Study

**DOI:** 10.2196/38474

**Published:** 2023-04-27

**Authors:** Isabel Klemme, Kamil J Wrona, Irja Marije de Jong, Christoph Dockweiler, Leona Aschentrup, Joanna Albrecht

**Affiliations:** 1 School of Public Health Bielefeld University Bielefeld Germany; 2 Athena Institute VU University Amsterdam Amsterdam Netherlands; 3 Faculty of Engineering and Mathematics Bielefeld University of Applied Sciences Bielefeld Germany; 4 Faculty of Health Bielefeld University of Applied Sciences Bielefeld Germany; 5 Department Digital Health Sciences and Biomedicine School of Life Sciences University of Siegen Siegen Germany

**Keywords:** people with type 1 diabetes, self-management, diabetes apps, vision assessment, anticipated stigma, qualitative research, Digitale Gesundheitsanwendungen, DiGA, mobile phone

## Abstract

**Background:**

Diabetes is a major global epidemic and serious public health problem. Diabetes self-management is a 24/7 challenge for people with type 1 diabetes that influences their quality of life (QoL). Certain apps can support the self-management of people with diabetes; however, current apps do not meet the needs of people with diabetes appropriately, and their safety is not ensured. Moreover, there are a multitude of hardware and software problems associated with diabetes apps and regulations. Clear guidelines are required to regulate medical care via apps. In Germany, apps must undergo 2 examination processes to be listed in the *Digitale Gesundheitsanwendungen* directory. However, neither examination process considers whether the medical use of the apps is sufficient for users’ self-management.

**Objective:**

This study aims to contribute to the technology development process of diabetes apps by exploring individual perspectives on desired features and content of diabetes apps among people with diabetes. The vision assessment conducted is a first step toward creating a shared vision among all relevant stakeholders. To ensure adequate research and development processes for diabetes apps in the future, guiding visions from all relevant stakeholders are required.

**Methods:**

In a qualitative study, 24 semistructured interviews with patients with type 1 diabetes were conducted, among whom 10 (42%) were currently using an app. To clarify the perceptions of people with diabetes regarding the functions and content of diabetes apps, a vision assessment was conducted.

**Results:**

People with diabetes have concrete ideas of features and content in apps to improve their QoL and allow them to live as comfortably as possible, such as informative predictions through artificial intelligence, improvements in signal loss and value delay through smartwatches, improved communication and information-sharing capabilities, reliable information sources, and user-friendly and discreet messaging options through smartwatches. In addition, according to people with diabetes, future apps should show improved sensors and app connectivity to avoid incorrect values being displayed. They also wish for an explicit indication that displayed values are delayed. In addition, personalized information was found to be lacking in apps.

**Conclusions:**

People with type 1 diabetes want future apps to improve their self-management and QoL and reduce stigma. Desired key features include personalized artificial intelligence predictions of blood glucose levels, improved communication and information sharing through chat and forum options, comprehensive information resources, and smartwatch alerts. A vision assessment is the first step in creating a shared vision among stakeholders to responsibly guide the development of diabetes apps. Relevant stakeholders include patient organizations, health care professionals, insurers, policy makers, device manufacturers, app developers, researchers, medical ethicists, and data security experts. After the research and development process, new apps must be launched while considering regulations regarding data security, liability, and reimbursement.

## Introduction

### Background

Diabetes mellitus is a major public health issue [1]. In 2045, a total of 700 million people worldwide will be affected by diabetes [2], whereas currently, 10% of the world population has type 1 diabetes (T1D) [3,4]. In particular, T1D care largely depends on self-management, which is a 24/7 responsibility that persists for 365 days per year [5,6]. The continuous challenge of monitoring blood glucose levels, correcting them with insulin, and finding metabolic balance reduces the quality of life (QoL) of people with diabetes [2,7]. Owing to the different pharmaceutical and supportive needs of people with diabetes [8], it is one of the most challenging health problems for the health care system and also a burden for people with diabetes themselves [7]. Numerous apps have been developed in the past years to support people with diabetes in managing their disease [6], and the importance of this topic has been confirmed by a growing body of literature [6].

Germany is the first country in which physicians can prescribe digital health apps [9]. In 2019, the Digital Supply Act entered into force in Germany. It allows physicians to prescribe apps that are listed in the *Digitale Gesundheitsanwendungen* (DiGA) directory by the *Bundesinstitut für Arzneimittel und Medizinprodukte*. Although there are 341,000 T1D cases [10] in Germany, no diabetes apps were registered in the DiGA directory while the study was conducted. The *Bundesinstitut für Arzneimittel und Medizinprodukte* decides via a fast-track procedure whether an app will be accepted into the DiGA [11]. However, whether the content and features of the app have sufficient medical benefits for the users is often still questionable. The legal conditions under which DiGAs are integrated into the DiGA directory place too little value on patient benefits [12]. A further prerequisite to be accepted into the DiGA directory is a Conformité Européenne medical product certification [12,13]. However, in this certification process, the completeness of the app is also not reviewed [14].

Despite the reported rapid growth of diabetes apps on the market [6], no diabetes apps were registered in the DiGA while the study was conducted, and numerous hardware and software problems need to be solved. First, apps do not fit into the daily activities of people with diabetes as most diabetes apps do not integrate the most important diabetes management tasks, such as physical activity, education, and health feedback [15]. In addition, the apps do not meet the needs of people with diabetes, such as user-friendly apps that provide actionable reminders and consolidate data across peripheral health devices [16]. Second, patient safety is not guaranteed as apps that manage the health of people with diabetes are mostly unregulated, do not ensure evidence of accuracy and clinical validity, show poor interoperability and standardization, and offer insufficient data security [6]. Therefore, the implementation of proper regulations is needed to ensure the medical use of apps and the security of the sensitive personal data of people with diabetes. They are highly interested in using diabetes apps that facilitate their self-management [17].

The main app features focus on nutritional intake, insulin injection, and physical activity [18], for example, via functions such as (1) diary of blood glucose measurements, (2) food database of carbohydrates, (3) bolus calculators for insulin dosage, (4) warning of too high or too low blood glucose levels, and (5) fitness trackers.

To avoid apps being certified and included in the DiGA directory whose features and content do not ensure medical use and are not optimally adapted to the needs of people with diabetes, this study aimed to contribute to the technology development process of apps by focusing on the perspective of people with diabetes. This should ensure that future apps that become available on the market and are possibly listed in the DiGA directory are optimally adapted to users’ needs and, thereby, to their self-management.

### Contribution of This Study to the Body of Literature

This study’s specific approach focused on the perspective of people with T1D and the improvement of their self-management via apps. The study responded to a research gap described in the literature: only a limited number of studies exist that have explored the use and feature preferences of people with diabetes concerning apps [16]. Furthermore, it is crucial to involve patients in defining the most important functions of apps [19] and have a better understanding of the potential of apps concerning self-management [15,20]. Future apps need more features to improve diabetic self-management [21,22] and must be tailored more to diabetic needs [23]. Thus, further research is needed on future diabetes app development [24].

### Contribution of This Study to a Wider Context

This qualitative study aimed to contribute to the technology development process of diabetes apps by exploring perspectives on the desired content and features of diabetes apps among people with T1D. This study was embedded in a complex and iterative process involving research, technology development, and implementation of new technologies in the health care sector. The specific objective was to contribute to the technology development process of diabetes apps by making explicit which features and content of apps can improve the self-management and QoL of people with diabetes.

In the context of health care, this study aimed to provide needs-based care for people with chronic diseases while ensuring maximum security (physical safety and data security). In the context of health economics, the aim was to reduce the costs of care for people with T1D by ensuring that optimized self-management results in fewer acute metabolic disorders with the need for hospitalization. The long-term goal was cost reduction via reduced and delayed comorbidities.

## Methods

### Overview

An exploratory study enabled the assessment of the perspectives of people with diabetes on self-management using diabetes apps [25]. In addition, a qualitative research study design fit the purpose of exploring the visions of people with diabetes concerning how diabetes apps can improve the diverse aspects of diabetes self-management. Qualitative semistructured interviews were conducted with patients with T1D who used an app and those who did not. Semistructured interviews offered the opportunity to gain a deep and contextualized insight into the content and features that participants would like to see in future apps.

### Theoretical Framework

To obtain people with T1D’s perspectives on the features and content of future diabetes apps, the Responsible Research and Innovation (RRI)—part of the European Framework Programmes—was chosen as the research approach. A vision assessment was used for this purpose.

The RRI approach aims to open up the research and innovation process to a wider group of stakeholders to “anticipate and assess potential impacts and societal expectations about research and innovation” [26]. In doing so, “all stakeholders involved should work together throughout the research and innovation process” [26].

**Figure 1:**
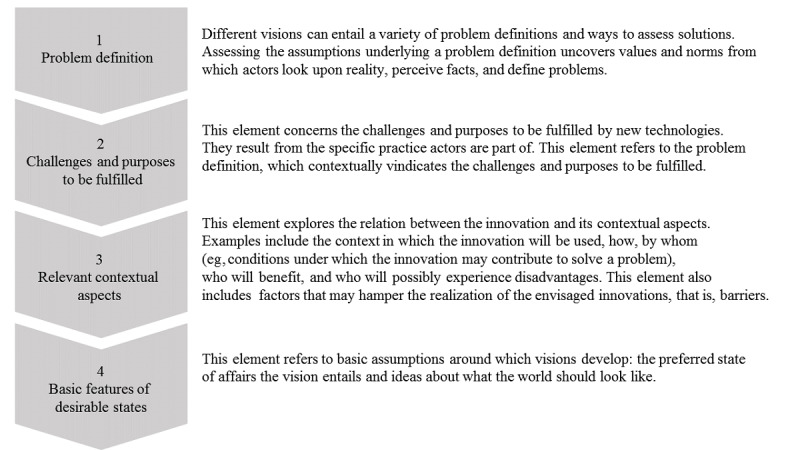
Vision assessment in the style of Arentshorst et al [26].

In line with the RRI, a vision assessment is used to actively include users’ perspectives in the technology development process. This can be done by making their visions explicit before the technology is developed. By opening up the technology development process to all relevant stakeholders and including their visions, important contributions can be made to this process [27]. By considering a wider set of facts and values associated with the technology in the making, its development process is rendered more likely to yield a responsible product. In this study, a vision assessment was conducted with people with T1D as, until now, their visions have not been sufficiently integrated into the research and technology development process of diabetes apps. A vision assessment provided an opportunity to explore the underlying assumptions about the expectations and concerns of people with diabetes. Moreover, it allowed for the early anticipation of potential negative unintended consequences. To date, only the vision of app developers has been considered in app development. Integrating the visions of people with T1D into the awareness-raising process opens the technology development process to a wider set of relevant stakeholders. Only when a vision assessment has been carried out with all relevant stakeholders can the guiding vision be merged into a shared vision [28]. A shared vision guiding technology development is more likely to attain societally beneficial products as it takes into account multiple perspectives. This study followed the 4 elements for a vision assessment developed by Arentshorst et al [28] (Figure 1) based on the studies by Fischer [29,30] and Chilvers and Kearnes [31].

The 4 elements provide insight into people with T1D’s views on what features and content of apps are necessary to optimize their self-management. The model has been validated for emerging technologies (sources). However, when applying this model in the context of diabetes, it is important to keep in mind that this study was not about the initial development of apps but about improving existing apps by adding new features and content. Therefore, the focus of this report was to identify a future guiding vision based on the perspective of people with diabetes that contributes to the technological development process of diabetes apps. Thus, the main research question was as follows: What are the perspectives of people with diabetes on the features and content of future diabetes apps?

The 4 elements of a vision assessment are translated into 4 subquestions: What is the problem definition in terms of relevant values and norms according to people with diabetes? What challenges and purposes are related to apps according to people with diabetes? What are the relevant contextual aspects of diabetes apps according to people with diabetes? What are the ideas of people with diabetes on what their world should look like?

### Procedure

The recruitment of participants took place from February 2021 to March 2021. A diabetologist was contacted to connect the researcher with potential interviewees among their patients. A purposive sampling strategy helps identify and select individuals who are especially knowledgeable about or experienced with the phenomenon of interest, in this case, diabetes self-management using apps [32]. Participants were included if they mastered the German or English language, had T1D, used or did not use a diabetes app, and were willing and able to agree to a voluntary consent form. Contact with potential participants was initiated with a personalized email request for a recorded interview with a short description of the research purpose and central topic. To explore a range of perspectives of people with diabetes, a large number of people with diabetes were interviewed, composed as heterogeneously as possible in terms of the apps, age, and sex. Table 1 shows the sociodemographic data (sex, age, and app) assessed through the questionnaire.

**Table 1:** Sample characteristics.

ID	Sex	Age (years)	Use of app
R01	Male	44	CGM^a^ but would prefer an app
R02	Female	32	CGM; prefers it over an app
R03	Female	43	FreeStyle Libre app ‬‬‬‬‬‬‬‬‬‬‬‬‬‬‬‬‬‬‬‬‬‬‬‬‬‬‬‬‬‬‬‬‬‬‬‬
R04	Male	81	CGM; has no smartphone
R05	Male	65	CGM; has no smartphone
R06	Female	36	Blood glucose self-monitoring; will switch to FreeStyle Libre app
R07	Female	78	Transplanted
R08	Female	32	FreeStyle Libre app
R09	Male	61	CGM; waits for a new sensor that can be connected to an app
R10	Female	47	CGM
R11	Female	40	FreeStyle Libre app
R12	Female	51	Transplanted; could not use sensor because of work condition
R13	Male	26	Blood glucose self-monitoring
R14	Female	42	FreeStyle Libre app
R15	Female	43	CGM but would like to use an app
R16	Male	25	FreeStyle Libre app
R17	Male	25	FreeStyle Libre app
R18	Female	24	No app but the patient gets a new insulin pump and then uses app
R19	Male	26	Dexcom app
R20	Male	23	FreeStyle Libre app
R21	Male	25	CGM; Dexcom has no compatibility with a phone but the patient wants to use the app
R22	Male	29	FreeStyle Libre app
R23	Male	28	CGM
R24	Female	24	FreeStyle Libre app

^a^CGM: continuous glucose monitoring.

In total, 24 individual semistructured interviews were conducted with people with diabetes based on a vision assessment until data saturation was attained. The decision on data saturation resulted from a consensus among the researchers. A total of 42% (10/24) of the participants used an app for their diabetes management. In total, 29% (7/24) wanted to use an app but were unable to do so because of circumstances beyond their control. The remaining 29% (7/24) of respondents did not want to use an app or had not considered it. Half of the participants (12/24, 50%) were female, and half (12/24, 50%) were male. Overall, participants were aged between 23 and 81 years. One-third of the participants (8/24, 33%) had experience with continuous glucose monitoring, and another third (9/24, 38%) had experience with the FreeStyle Libre app. Overall, 62% (15/24) of the interviews were conducted at a diabetologist practice face to face, and 38% (9/24) of the interviews were conducted via videoconference because of the restrictions of COVID-19. A data management plan was created and served as a guide for this research and the data collection process.

### Analysis

Qualitative analysis was performed by IK in an iterative and nonlinear manner using the general steps of qualitative data analysis described by Creswell [32]. This was supported by ATLAS.ti (ATLAS.ti Scientific Software Development GmbH), a specialized computer software for qualitative research [33]. First, inductive content analysis was applied to organize and analyze the raw data through an open coding process (Multimedia Appendices 1 and 2). Subsequently, these codes were checked against the codebook and deductively created from the theoretical framework displayed in Figure 1 (deductive coding). Newly emerging codes were added, or existing codes were amended to allow for the inclusion of new insights. Axial coding was carried out to make connections between codes, determine relationships, and form a hierarchy among codes. Through this process of categorization, the final broader analytical themes were formed. The inductive coding process did not expand the broader analytical themes of the theoretical framework used (Textbox 1). Overall, the coding procedure used a combination of emerging and predefined codes [34].

**Definitions of the final analytical themes.**

- Problem definition: underlying problems that people with diabetes experience with their disease
- Challenges: problems that people with diabetes experience in self-management using apps
- Purposes: features that future apps need to entail to solve these problems
- Relevant contextual aspects: app characteristics [3] and technical aids such as apps [28]
- Desirable states: the ideas of people with diabetes about what the world should be like

### Ethical Considerations

Before starting the recruitment of participants, a research ethics self-check provided by the Vrije Universiteit Amsterdam Faculty of Science [35] was conducted that stated that this study did not require approval from a medical ethics committee.

Before the interviews, participants were informed about the study’s purpose and the right to withdraw at any time. They received information from the diabetologist or via email regarding the research objectives and the confidentiality of their personal information. Written or verbal voluntary consent for the recorded interviews was asked for and obtained before the interviews began. Interviewees were allowed to refuse to answer questions or provide sensitive information and end the interview at any time. The interview design was based on the operationalization of the 4 subquestions. The interview guide included a topic list with possible probing questions. The duration of the interviews ranged from 45 minutes to 1 hour. Notes were taken during and after the interviews.

The interview recordings were transcribed verbatim with the participants’ permission. To ensure the privacy of the interviewees, the researcher was the only one with access to the recordings. The audio recordings were deleted after transcription, and the anonymized transcripts are stored securely for 5 years.

## Results

### Overview

Henceforth, a distinction will be made between *users* (current app users) and *interviewees* (all interviewed participants). The collected data showed that the main reason why people used an app for their diabetes management was to improve daily self-management. Within the interviews, age, technical understanding, usability of the sensor (which is attached to the upper arm), work conditions, and compatibility with the physician’s practice could be identified as factors influencing the current use of apps. There were no identified differences in the answers from participants of different sex.

Analysis of the interviews resulted in the identification of one guiding vision on diabetes apps from the perspective of people with diabetes. Diabetes apps are envisioned to improve people with T1D’s QoL and enable them to live as normally as possible without anticipating stigma.

**Figure 2:**
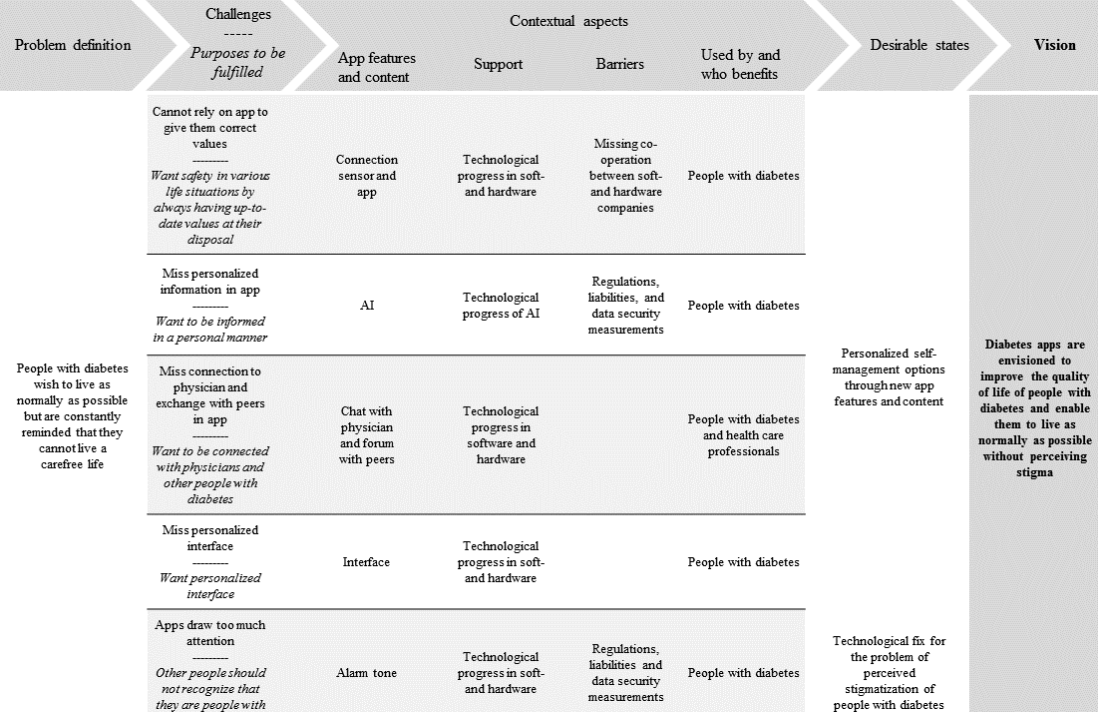
Results of the vision assessment: elements constructing the vision on diabetes apps from the perspective of people with diabetes. AI: artificial intelligence.

An overview of the results is presented in Figure 2. The *Results* section is structured in line with the elements displayed therein.

### Problem Definition and Underlying Values

The main problem that people with T1D experience with their diabetes is that they wish to live as normally as possible but they are constantly reminded that they cannot live a carefree life:

I think as a person with diabetic you are always between the two poles of absolute control, and I do not care, I always oscillate between these two poles, and you never reach one of them, you are always in between.

R10; female; aged 47 years

This is related to the ambivalence that people with diabetes must endure every day concerning their health. People with diabetes reported not feeling sick yet having to manage a chronic disease 24/7:

Diabetes is not a real disease in comparison to cancer or dementia, because you can do everything you want.

R22; male; aged 29 years

Values such as self-determination, individuality, privacy, and safety, as well as associated norms, are subject to the aforementioned ambivalence between feeling healthy and constantly managing their diabetes. For people with diabetes, the value of *self-determination* means that they can live a self-determined life in which they have freedom of decision and action. However, in everyday life, they face problems with their blood glucose levels that make it impossible for them to have complete freedom of decision and action. The value of *safety* is important as people with diabetes live with a constant fear of hypoglycemia, which can have deadly consequences. Thus, they need regular and reliable information on their current blood glucose levels. *Individuality* about diabetes means that people with diabetes do not feel individually supported by their apps as these are not tailored to their life circumstances. Moreover, people with diabetes attach great importance to the value of *privacy*. However, it is important to note that this value is related to the wish not to attract attention to their disease in everyday life situations rather than to concerns about potential access to personal health data generated through apps:

A more discrete sound would be great so not everyone notices it and it does not attract everyone’s attention.

R16; male; aged 25 years

### Challenges of Current Apps and Purposes to Be Fulfilled by New Apps

Challenges were reported when using apps for self-management. These challenges resulted in the purposes that people with diabetes would like to see in future apps. First, apps were found to attract too much attention from other people. Therefore, future apps, or rather the use of these apps, should not attract the attention of other people so that people with diabetes do not have to anticipate stigmatization:

During n school exam, it is very annoying when the App makes sounds because the professors might think you are cheating.

R18; female; aged 24 years

Second, people with T1D could not rely on the reality of the values displayed in the app. Therefore, people with diabetes wished that future apps display real-time values that they can rely on without any delay. Third, people with diabetes missed reliable, solid, and personalized information in the apps. Thus, they wanted to be informed in a personalized and high-quality manner by future apps continuously. Fourth, they missed personal exchange options with physicians and other people with diabetes. Hence, they desired the opportunity to communicate with physicians and other people with diabetes via apps:

It would be great to quickly get the information you need from your doctor or other people with diabetes, especially in tricky or new situations.

R21; male; aged 25 years

### Relevant Contextual Aspects of Diabetes Apps

Interviewees reported that the following existing app features and content need to be improved to optimize their self-management: alarm tone, sensor, and app connection. Moreover, interviewees reported that the following new app features and content need to be developed to optimize their self-management: artificial intelligence (AI), information, and communication features.

#### Alarm Tone

Users reported ambivalence toward the alarm tone. It was reported to be both the most useful feature to prevent hyper- or hypoglycemia and the most annoying feature. Most users (9/10, 90%) reported missing features to individually select the alarm tone and decide when to turn it on and off. The alarms were reported to be the most indiscrete feature as they attract a lot of attention from other people, leading to anticipated stigmatization:

When I am at work, I do not want my colleagues to notice the sounds of the App. The App makes sounds when I hypoglycaemia. They might think that I am weak or that I cannot concentrate any longer.

R14; female; aged 42 years

The interviewees especially expressed this in situations such as being at work or at a restaurant or engaging in sports. In addition, waking up to a harsh tone every night was a reason why people with diabetes turned off the alarms at night, which means that they accepted the risk of hypoglycemia. However, other users said that the alarm during the night was the greatest feature of the app as it made them feel safe. Users reported appreciating a feature that would let them individually select the alarm sounds and time slots when it is on or off. Most interviewees (17/24, 71%) wished for an installation of an app on a smartwatch. A vibrating smartwatch was argued to be superior to an alarm tone. In this way, the potential of anticipated stigmatization would also be minimized:

I am already wearing a smartwatch and it vibrates when I get a message. It is inconspicuous and discreet, and not everyone notices it. Also, a quick look at the watch and briefly holding the watch in front of the sensor is more discreet.

R15; female; aged 43 years

#### Sensor and App Connection

Concerns regarding delayed value transmission and signal loss were widespread among interviewees. All users (10/10, 100%) stated that delayed value transmission hampered their self-management:

I always have to control the system and listen to my body and compare how I feel to what the App says. This is because the sensor measures the concentration of glucose in interstitial fluid which has a time lag of sometimes 20 minutes compared to blood glucose measurements.

R16; male; aged 25 years

Some interviewees (3/24, 13%) even reported not using an app as they could not handle the value delay. The delayed values reportedly made them insecure. Thus, they wished for clear information about value delay in the app:

If the values always have a delay of 20 minutes, then it should be written down for you like 20 minutes ago this was your value. But it is not displayed anywhere, you just have to know it.

R17; male; aged 25 years

Furthermore, missing values because of signal loss between the app and the sensor were mentioned repeatedly:

If you leave the phone somewhere then the value transfer from the sensor to the App does not work anymore, and you cannot see your values. This is very annoying.

R2; female; aged 32 years

Moreover, some interviewees (6/24, 25%) stated that an improved connection between apps and sensors from different providers would greatly increase individual self-management. Most interviewees (17/24, 71%) preferred an app installation on a smartwatch that diminished the problem of signal loss as the distance between the sensor and the watch is shorter. The combination of improved value display and reduced signal loss was said to increase the usability of the app and, thereby, highly improve the daily self-management of people with diabetes.

#### AI Features

Many users (7/10, 70%) stated that they adapted to the app and not the other way around:

The users adapt to the system and not the system to the user.

R16; male; aged 25 years

All users (10/10, 100%) indicated that apps lack sufficiency in ensuring good self-management that suits their daily activities. Concerning stressful situations at work, some interviewees (8/24, 33%) complained that they forgot to check their values and, thus, forgot to inject insulin for 2 reasons. First, they did not have their phone or blood glucose meter device with them (eg, when a phone is not allowed at work). Second, blood level measurement attracts too much attention from colleagues. Some users (3/10, 30%) stated that they turned the alarms off at work so that other people would not ask questions or notice them:

I am a teacher and during my break, I always leave the break room to check my glucose levels. I do not want other people to notice. Also, during meetings, I switch off the sound because I do not want others to think that I cannot concentrate any longer.

R14; female; aged 42 years

Interviewees expressed the wish for an AI on a smartwatch app that reminds them to check their values or shows them the prognosis of their blood glucose level.

In addition, during activities such as sports or eating at a restaurant, users reported that current apps are not well adapted to their needs. Some interviewees (5/24, 21%) stated that they did not try a new sport as they were afraid that they could not handle their self-management before, during, and after the new activity. Interestingly, most user (8/10, 80%) reported missing profound personal information about sports in the app (content). They suggested that algorithms and AI should provide hints or reminders regarding nutrition, insulin dosage, and sports. The AI should recognize patterns of body stature and fitness level to provide users with predictions, such as a graph in which one can see the predicted blood glucose level for a certain sport:

It could predict blood glucose level for sports. So that I can see how the values will change during the activity and I can adapt my eating behaviour and activity to it.

R13; male; aged 26 years

Furthermore, interviewees repeatedly reported that a prerequisite for that is that diabetes apps are connected to other apps:

Prediction is not working at all at the moment. They should connect the Apple Health App to the diabetes App and then tell me how much insulin I have to inject. Or when I do sport the App could give me the alarm a bit earlier and tell me that the values will go down quicker.

R17; male; aged 25 years

Some interviewees (3/24, 13%) said that, in the beginning phase, they took a scale with them to restaurants to calculate insulin injections, which was reported to be an uncomfortable situation in terms of anticipated stigmatization. Most interviewees (17/24, 71%) expressed that a photo AI calculating the bread unit (BU) would be easy to integrate into their daily lives and be more discrete at a restaurant. Newly diagnosed interviewees were unanimous in the view that this feature would be one of the best features to alleviate their daily lives.

#### Information and Communication Features

A recurrent theme in the interviews was that individually tailored, in-depth, and informative content was missing in the current apps. In unknown and challenging situations, most interviewees (17/24, 71%) reported missing well-founded sources offering reliable and individual information in apps, which could increase safety in everyday life. For example, interviewees expressed the wish for more information in the form of notifications for activities such as sports, traveling, or consuming alcohol:

It would be useful to enter interests in the App like Pinterest and thereby get individually tailored information via notifications.

R17; male; aged 25 years

In addition, communication was expressed to be important but still missing. Some interviewees (11/24, 46%) reported that information exchange and connection with peers were extremely important when they were first diagnosed. Communication features such as chats and forums were stated to enable enhanced information exchange possibilities. Most interviewees (13/24, 54%) preferred a direct chat with their physician over a chatbot to receive timely information in tricky situations. A chat function with their physician was preferred over a chat with peers:

I would only like to have a quick contact with my doctor. He knows my history and I trust him.

R19; male; aged 26 years

Half of the interviewees (12/24, 50%) considered a forum feature to be more suitable for their daily life than a direct chat with peers. Furthermore, some interviewees (5/24, 21%) were aware that it is difficult to ensure that the available information in forums is medically accurate, trustworthy, and up-to-date.

#### Supporting and Hampering Contextual Aspects

A range of contextual aspects were mentioned by most interviewees (18/24, 75%) that support or hamper the realization of future apps features. On the one hand, the main supporting aspects mentioned were fast technological innovation progress, digitalization, and AI. These were said to improve app features and their compatibility with other devices (smartwatches). On the other hand, interviewees perceived the lack of collaboration between software and hardware companies as a barrier to the implementation of innovations. In addition, many interviewees 10/24, 42%) mentioned that data security measures hamper physician practices in transferring data from the app directly into their professional software. This was reported as most likely to remain a barrier in the future. Interviewees were aware that integrating AI and the other aforementioned features into future apps will take some time as aspects such as regulations, liability issues, and data security measures are barriers to the implementation of medical devices.

Interestingly, interviewees did not attach high importance to the data security of the aforementioned new features. Most interviewees (17/24, 71%) stated that they did not care about data security, especially if this hampers the introduction of the features that would make their self-management easier:

When the added value of the feature outweighs the supposed disadvantages, I would like to use the new App features.

R18; female; aged 24 years

Moreover, most (10/17, 59%) assumed that they were the only ones thinking that way and stated that most likely others would give a higher priority to data security than they did:

I personally don’t see blood glucose level as super sensitive data, but most likely other people may see it quite differently.

R17; male; aged 25 years

Some interviewees (8/24, 33%) stated that they believed that, if somebody wants to find out things about them, they will. Some respondents (7/24, 29%) also stated that they may not have an overview of the extent to which others could harm them by accessing their personal data.

### Desirable States of People With Diabetes

The analysis revealed 2 desirable states, from which the guiding vision was developed. According to the interviewees, the combination of both states leads to an improvement in their QoL and the feeling of living a normal life. Thus, the interviewees formulated the following ideas about what the technical solutions should look like: (1) personalized self-management options with new app features and content and (2) a technological fix for the problem of anticipated stigmatization of people with diabetes.

First, all the features and contents named by the interviewees optimized their self-management. Interviewees stated that all the mentioned features and contents could lead to a more personalized app that adapts to their personal and medical needs. In particular, algorithms and AI could provide them with individually tailored information in the form of tips, reminders, and predictions for a wide range of activities. Features and contents of future apps were reported to open up options to combine the app with a variety of hardware devices, receive individually tailored information, and communicate with health professionals and peers. According to interviewees, all these features and contents of future apps could lead to an optimized personalized self-management.

Second, some of the features that interviewees reported should be included in future apps to minimize anticipated stigma. These are features to individually select alarm tones and time slots when the alarm is on or off, as well as the possibility to install an app on a smartwatch that vibrates and does not make any noise and, therefore, cannot be perceived by others. The possibility to take a photo of the food to estimate BUs was also mentioned. These features make sure that others do not recognize them as people with diabetes and, thereby, reduce the belief of people with diabetes that others stigmatize them as sick.

From these 2 desirable states, the guiding vision was developed. According to the interviewees, only the combination of both leads to an improvement in QoL and living as normally as possible without anticipating stigma.

## Discussion

### Principal Findings

#### Overview

From the perspective of people with T1D, diabetes apps can improve QoL and enable them to live as normally as possible without anticipated stigma. This guiding vision is connected to two aspects: (1) the need for personalized self-management options through new app features and content and (2) the need for technological fixes regarding the issue of anticipated stigmatization generated through the sounds and visibility of self-management activities. In this regard, people with diabetes have a clear idea of which features and contents should be included in future apps to improve self-management. Overall, six main topics surfaced: (1) improvement of the sensor and app connection via smartwatch, (2) innovation of blood glucose measurement methods, (3) prediction via AI, (4) information and communication via forum and chat, (5) alarm tone reduction by using a smartwatch, and (6) data security.

#### Improvement of the Sensor and App Connection via Smartwatch

Signal losses when connecting the sensor and app can be reduced by using a smartwatch as an in-between solution. The distance between the sensor and the app when installed on the smartwatch is reduced. However, a lack of cooperation between companies, liability issues, and data security measures were mentioned as possible reasons why only a few companies offer this solution, which is also confirmed by Freckmann [36]. Participants named data security measures as the main reason hindering the realization of a sensor-smartwatch connection. This finding was also confirmed by Cohen [37], who further states that medical device regulatory procedures are a general barrier to implementing new technologies. Overall, few authors have mentioned smartwatches for diabetes management support. For example, Årsand et al [38] concluded that smartwatches make it easier for people with diabetes to record, monitor, and analyze their blood glucose levels in everyday life. However, so far, users still need a smartphone as an intermediary to receive the data on a smartwatch. Moreover, widespread adoption will only be possible when the aforementioned hindering measures and regulatory processes are alleviated.

#### Innovation of Blood Glucose Measurement Methods

App users demanded that apps note that the displayed values of blood glucose are delayed. Current sensors have a time lag of 5 to 20 minutes [39]. Aware of this issue, manufacturers are currently developing new ways to measure blood glucose levels to improve delayed value transmission, such as mini-sensors that can be implanted under the fingertip, sensor tattoos, or a tool that detects blood glucose levels in exhaled air [40]. Moreover, research is also being conducted on how smartwatches can directly measure blood glucose levels and transmit them in real time [41].

#### Prediction via AI

The research results show that predicting blood glucose levels through algorithms and AI might improve self-management of various daily activities. However, Kriventsov et al [39] argue that the challenge in AI prediction is the value lag. Nevertheless, Kriventsov et al [39] also state that it is possible to accurately predict blood glucose fluctuations using personalized machine learning models [39]. These findings are consistent with those of Fatehi et al [42], who found that machine learning algorithms and AI can provide personalized predictions based on data models from similar cohorts and patient histories. Therefore, using AI in future apps to accurately predict blood glucose levels would greatly improve the self-management of people with diabetes in everyday life.

In addition, Maharjan et al [43] proposed an AI-driven personalized diabetes virtual assistant to support people with T1D in their daily activities. The AI could then learn from the user’s habits, inputs, or questions so that the user receives better support over time [43]. For example, Vettoretti et al [44] developed an AI algorithm that can predict future blood glucose levels based on past glitches and errors. The developed algorithms learn from the user’s collected data (amount of exercise, sleep, and stress) and integrate them into the prediction models [44]. However, Kulzer [45] stated that the capacity of AI models for decision-making, data security issues, and liability issues are limited when using AI. This statement is consistent with the barriers identified by respondents in our study. Moreover, the intense societal debate on the opportunities and risks of AI, including ethics, liability, and data protection, must be addressed [45].

Finally, the participants desired a photo AI function to help calculate their BUs. An AI currently in development by the start-up SNAQ, for example, performs an image-based nutritional analysis of meals and calculates the necessary insulin dosages [46]. Another example is an AI for insulin optimization that can predict the blood sugar level for the next 60 minutes developed by the start-up Hedia. This AI also detects eating habits over time and suggests an adjustment tailored to the individual [47].

#### Information and Communication via Forum and Chat

The interviewees appreciated the possibility of information exchange with peers via forums, although they were also aware that it might be difficult to ensure profound knowledge in these forums. In accordance with our findings, a study by Jeon and Park [48] showed that participants’ self-care motivation significantly improved by offering a bulletin board in their app where people with diabetes could share their experiences and express empathy with others.

A cross-sectional study by Litchman et al [49] suggested that individuals who participate in web-based communities are more likely to have improved blood glucose levels and better QoL. Despite the proven positive effects, Eberle and Ament [50] state that social features such as forums are not yet present in many apps. Hence, additional research is needed to explore how forum use influences users’ self-management [50]. Furthermore, research will be needed to determine how engagement in these forums may affect health outcomes [49].

Our research has also shown that people with T1D wish for a chat feature in the app to communicate with their physicians about certain or urgent problems. They did not want to communicate with other physicians or a chatbot. The findings of Zhang et al [51] are similar to our research. Moreover, the participants stated that the feedback has to be on time to be useful. However, surprisingly, a report by the Center for Advanced Hindsight found that people with diabetes accepted a chatbot as an expert in a certain domain, and over time, they became attached to the bot as a friend. The reason for this is that a chatbot can provide continuous and reliable support to the user in a tailored manner [51]. The outcomes of the report by the Center for Advanced Hindsight [52] are somewhat contrary to our findings. Therefore, more research is needed to assess whether people with diabetes would prefer a chat with their physician or a chatbot and how either of these solutions can be integrated into the apps to become a reliable source of information. In this regard, it is to argue whether the findings are not necessarily contradictory but complementary in the sense that, if a chat with a physician is not possible, people with diabetes will probably settle for a chatbot, which could be satisfying enough for them. For some people, it could also be a barrier to approaching a physician with a (more or less trivial) question concerning their health, and as a consequence, these people are more comfortable asking a chatbot.

#### Alarm Tone Reduction While Using a Smartwatch

App users criticized the sound of the alarm tone of apps while using the smartwatch, which easily attracts the attention of other people. Thus, they wanted a technological fix to minimize their fear of judgment from others (anticipated stigmatization). In this context, the study by Brazeau et al [53] confirms that noticeable equipment leads to the fear of being judged by others, which is more pronounced among young people and is associated with lower diabetes-related self-management and self-efficacy, severe hypoglycemia, and diminished sense of well-being. Furthermore, the cross-sectional study by Gredig and Bartelsen-Raemy [54] found that anticipated stigma has a negative impact on QoL, which is in line with our findings.

Moreover, participants of the studies by Gredig and Bartelsen-Raemy [54] and Liu et al [55] expressed the wish for education for the general public to reduce diabetes stigma. Furthermore, Gredig and Bartelsen-Raemy [54] concluded that health care professionals play a key role in changing the stigma about diabetes, and the authors suggested that health campaigns for diabetes could be developed similarly to HIV campaigns. However, to tackle stigma at its roots, a combination of education of the public, health campaigns, and technology development could be a holistic approach to tackle the fear of being judged by others.

#### Data Security

Another key aspect was data security, which involves safely managing the information of app users. However, interestingly, our interviewees did not attach high importance to the data security of new app features when the added value of a new feature was high for their self-management. The fact that interviewees did not attach high importance to data security is probably due to a lack of experience with adverse events such as security breaches or hacker attacks. Still, according to Britton and Britton-Colonnese [56], the safety of people with diabetes is at risk when cybersecurity is not achieved. Filkins et al [57] listed the implications that data breaches can have for people with diabetes. For example, malware can be installed, which leads to both loss of control of the device and corrupted data, with severe consequences for people with diabetes. In conclusion, people with diabetes have to be made aware of these dangerous consequences.

Moreover, Britton and Britton-Colonnese [56] and Fleming et al [6] revealed that people with diabetes are often unaware of what they authorize when they install an app. In addition, when they want to use a certain app, they have to accept how manufacturers gather, share, and use their data [56]. These findings support the need for more digital literacy.

### Limitations

This study has limitations resulting from people with T1D and the apps that they currently use or used in the past. Only the guiding vision of 1 stakeholder group was assessed and not those of various other relevant stakeholders. This study was limited by the use of only interviews because of COVID-19 restrictions. Focus groups are more suited for the construction of a guiding vision as people do not develop visions or opinions in isolation but through discussion and interaction with their surroundings. Focus groups are better able to mimic this process.

Although the interviewees who did not use apps only had limited imagination about the potential of apps, some interviewees (4/24, 17%) only used an app for some time as they had problems with the sensor app system and stopped using it (eg, an allergic reaction to sensor glue). This limited their imagination because of negative attitudes toward technological devices.

Furthermore, this report did not focus on all the systems that are available for diabetes self-management. Examples of what was not considered in this report are insulin pumps and automated insulin delivery systems. Finally, interviewees used different apps. Some used apps in connection with a sensor, and others used apps with a sensor and pump.

### Conclusions

People with T1D have a specific idea of the features and content needed in future apps to improve their self-management—and, thereby, their QoL—and enable them to live as normally as possible without anticipating stigma. These include personalized predictions via AI, improvements in signal loss and value delay via smartwatch, improved communication and information exchange possibilities (chat and forum), profound information sources, and warning through a vibrating smartwatch.

The vision assessment conducted in this study is a first step toward creating a shared vision of all relevant stakeholders for the technology development process of diabetes apps. Therefore, the next step could be to assess the guiding visions of the relevant stakeholders included in the research and technology development process. Thereby, their visions are made explicit and can be integrated into a shared vision. The relevant stakeholders that should be included are patient organizations; health care professionals (diabetologists, diabetes advisers, and dieticians); insurers; policy makers; device manufacturers; app developers; researchers; medical ethicists; and medical data security specialists. This enables the development process of future apps to be guided by a rich and shared vision of all relevant stakeholders, thereby raising the likelihood of the apps being societally relevant and responsible. The research and development process is followed by the implementation of new apps. The implementation requires regulations regarding data security (AI), liability issues, and reimbursement (the Digital Supply Act).

## Appendix

Multimedia Appendix 1 Coding tree.

Multimedia Appendix 2 COREQ (Consolidated Criteria for Reporting Qualitative Research) checklist.

## Abbreviations

AI: artificial intelligence
BU: bread unit
DiGA: Digitale Gesundheitsanwendungen
QoL: quality of life
RRI: Responsible Research and Innovation
T1D: type 1 diabetes
