# Supplementary material for: Integration of the Vision of People With Diabetes Into the Development Process to Improve Self-management via Diabetes Apps: Qualitative Interview Study
Source: JMIR Diabetes. 2023 Apr 27;8:e38474. doi: 10.2196/38474 (PMC10176130; doi:10.2196/38474)
Supplement: Multimedia Appendix 1 [file diabetes_v8i1e38474_app1.docx]

| **Coding theme** | **Coding subthemes** | **Origin** | **Definition** | **Examples** | **Notes** |
| --- | --- | --- | --- | --- | --- |
| Problem definition | Improve Quality of life | Inductive | Improve quality of life to the point where you can live life like not having diabetes. (14.05.2021) | “To me this app improves my quality of life immensely.” (TRANSCRIPT_22) | Seen in all transcripts. For both people who use an app already and people who do not. |
| Problem definition (Values) | Individuality | Inductive | In this context individuality means that app features have to adapt to the user. (16.05.2021) | “I want to decide and choose features individually.” (TRANSCRIPT_3) | This is mentioned by all interviewees. I recognised it when coding transcript number 3. |
|  | Privacy | Inductive | In this context privacy means that app features have to be discrete in order for users to enjoy using the app for their self-management. (20.05.2021) | “A more discrete sound would be great so not everyone notices it and it does not grab everyone’s attention.”  (TRANSCRIPT_16) | This was a very obvious value. Every interviewee mentioned this with regards ot the alarm tones. |
|  | Safety/ security | Inductive | In this context security has two meanings. First security with regards to their diabetes and blood glucose levels and second the topic of data security. (16.05.2021) | “I don’t think that data security is an issue or privacy. I am not worried about that.”  (TRANSCRIPT_2) | I was surprised about the answers with regards to the second meaning. The different topics of security emerged early in the coding process. |
|  | Self-determination | Inductive | In this case self-determination means that they can live a self-determined life in which they have freedom of decision and action. (22.05.2021) | “I don’t want to have more things I have to do or where I am told what to do.”  (TRANSCRIPT_10)  Especially, during the night the alarm tone makes me feel more safe.  (TRANSCRIPT_13) | This was an obvious value which all interviewees mentioned. This underlies the wish to improve QoL. |
| Challenges and purposes | Correctness of values | Inductive | Blood glucose levels are often delayed this hampers self-management. (14.05.2021) | “They could at least state in the app that the values are delayed.”  (TRANSCRIPT_17) | This challenge was mentioned by most diabetics. Some so not use apps because of this. |
|  | Exchange options to receive information | Inductive | Diabetics would like to receive more information and be in contact with doctors in peers more often. (21.0502021) | “The idea of a chat or forum with other peers is a great idea to exchange experiences and knowledge.”  (TRANSCRIPT_13) | Connectivity was already discovered in the second interview. Different aspects of connectivity were found out throughout the coding process. |
|  | Personalised interface | Inductive | In this report usability of the app is meant. The usability of an app is crucial for the users and one important point is the user friendliness of the interface.  (22.05.2021) | “The apps interface has to better and the app should adapt to me and not the other way around.”  (TRANSCRIPT_16) | The apps usability has to be improved in many different ways. I noted that during coding. New issues with regards to usability came up and also similar ones were mentioned. |
| Relevant contextual aspects | Too much attention grabbed by sounds (Alarm tone feature) | Inductive | Too much attention of other people is meant here. The alarm tones of the current apps grab too much attention of other people. (20.05.2021) | “I do not want others to notice that I have diabetes. I turned all alarms off”  (TRANSCRIPT_4) | Different findings for the alarm tone. Some said it’s a great feature other say it is annoying. |
|  | Sensor and app connection feature | Inductive | The connection is between the CGM sensor and the app. The sensor measure blood tissue glucose which is not the real blood glucose. Thus values are delayed. (18.05.2021) | “The value is always delayed you have to know it they do not say it in the app. This makes self-management complicated.”  (TRANSCRIPT_16) | This became apparent in all interviews with people who use a sensor. Some do not use a sensor because of that. |
|  | Design of interface | Inductive | The interface is not user-friendly enough. So easy of use is not given.  (19.05.2021) | “I have to use the same app interface as my grandma”  (TRANSCRIPT_17) | This was an interesting finding for me. Since it is no direct feature, but the general usability of current apps is low. |
|  | Miss personalised information  Artificial intelligence | Inductive | In this case AI should predict blood glucose levels for diabetics and give diabetics personalised information.  (15.05.2021) | “It would be great to see predicted blood glucose levels before I want to do a sport.”  (TRANSCRIPT_13) | This was most often mentioned with regards to sports or eating. |
|  | Information and communication features | Inductive | Missing information and communication with the doctor and peers were mentioned.  (18.05.2021) | “It would be great to have a chat function so I can ask questions in new and tricky situations”  (TRANSCRIPT_07) | A chat with a doctor and a forum to exchange information with peers was most often mentioned. |
| Supporting aspects | Technological innovation, digitalisation | Inductive | Innovations like AI and smartwatch combination with app were meant by that. (19.05.2021) | “A smart watch would be great. I already have one.”  (TRANSCRIPT_12) | Most often the wish for smartwatch connection and integrated AI were mentioned innovation sin the near future to support their self-management. |
| Hampering aspects | Missing cooperation between soft- and hardware companies | Inductive | More connection and cooperation of soft -and hardware companies was wished for. So a smartwatch could be integreated in the self-management.  (20.05.2021) | “If combability of the sensor, phone and app would improve that would be really great.”  (TRANSCRIPT_21) | The cooperation of companies would give people more options and would make the app even more individual. |
|  | Regulations, liability issues, data security | Inductive | Laws, data security measures and liability questions were said to be hampering the introduction of many new app features and the combination with smartwatches. (20.05.2021) | “ I think companies are afraid also to get sued when things go wrong or the AI predictions is false.”  (TRANSCRIPT_17) | Strict regulations were mentioned many times to be barriers for the introduction of new features in the apps. |
|  | Data security | Inductive | Diabetics do not attach high importance to data security. (20.05.2021) | I personally do not mind it does not really matter to me. I would like to use features that support my self-management.  (TRANSCRIPT_02) | This topic became apparent in the second interview already and was confirmed by all other interviews. |
| Desirable states | Self-management | Inductive | The improvement of their self-management is crucial to improve their QoL. (22.05.2021) | “The app greatly enhances my daily self-management already.”  (TRANSCRIPT_16) | Self-management is really a 24/7 task and can be improved by man new app features. |
|  | Perceived stigma | Inductive | Diabetics think that others think badly of them when other hear the alarm sound or see the sensor.  (19.05.2021) | “I do not want others to see or notice that I have diabetes at sports this is embarrassing.”  (TRANSCRIPT_11) |  |
| Vision | Live as normal as possible | Inductive | In this case it means that diabetics want to minimize the thoughts and tasks they have to execute on a daily basis to manage their diabetes.  (23.05.2021) | A closed loop system is also interesting, and it is great to see how people can perfectly live with their diabetes.  (TRANSCRIPT_19) | This was found to be the main vision of all people with diabetes. All other codes are leading towards this main vision. Not all expressed it directly, but most interviewees made it clear more indirectly that this is their overall vision. It took some time to find it out. |
